# Systematic Alignment Analysis of Neural Transplant Cells in Electrospun Nanofibre Scaffolds

**DOI:** 10.3390/ma16010124

**Published:** 2022-12-23

**Authors:** Aina Mogas Barcons, Farhana Chowdhury, Divya M. Chari, Christopher Adams

**Affiliations:** 1 Sheffield Institute for Translational Neuroscience, University of Sheffield, Sheffield S10 2HQ, UK; 2 Labcorp Drug Development, Otley Road, Harrogate HG3 1PY, UK; 3 School of Medicine, Keele University, Newcastle-under-Lyme ST5 5BG, UK; 4 School of Life Sciences, Keele University, Newcastle-under-Lyme ST5 5BG, UK

**Keywords:** neural tissue engineering, electrospinning, neural stem cell, stem cell transplantation, image analysis

## Abstract

Spinal cord injury is debilitating with functional loss often permanent due to a lack of neuro-regenerative or neuro-therapeutic strategies. A promising approach to enhance biological function is through implantation of tissue engineered constructs, to offer neural cell replacement and reconstruction of the functional neuro-architecture. A key goal is to achieve spatially targeted guidance of regenerating tissue across the lesion site to achieve an aligned tissue structure lost as a consequence of injury. Electrospun nanofibres mimic the nanoscale architecture of the spinal cord, can be readily aligned, functionalised with pro-regenerative molecules and incorporated into implantable matrices to provide topographical cues. Crucially, electrospun nanofibers are routinely manufactured at a scale required for clinical use. Although promising, few studies have tested whether electrospun nanofibres can guide targeted spatial growth of clinically relevant neural stem/precursor populations. The alignment fate of daughter cells (derived from the pre-aligned parent cells) has also received limited attention. Further, a standardised quantification methodology to correlate neural cell alignment with topographical cues is not available. We have adapted an image analysis technique to quantify nanofibre-induced alignment of neural cells. Using this method, we show that two key neural stem/precursor populations of clinical relevance (namely, neural stem cells (NSCs) and oligodendrocyte precursor cells), reproducibly orientate their growth to aligned, high-density electrospun nanofiber meshes, but not randomly distributed ones. Daughter populations derived from aligned NSCs (neurons and astrocytes) maintained their alignment following differentiation, but oligodendrocytes did not. Our data show that pre-aligned transplant populations can be used to generate complex, multicellular aligned-fibre constructs for neural implantation.

## 1. Introduction

Spinal cord injury (SCI) can be severe and debilitating, affecting around 500,000 people in the world every year [1], with attempts at repair so far ineffective. A major clinical goal is to develop regenerative strategies which can overcome the growth-inhibitory nature of the injury and restore the cells and architecture of the spinal cord. Advanced tissue-engineered scaffolds provide a platform to achieve such a therapy by their ability to mediate multiple reparative processes, such as, (i) stem cell incorporation and protection to improve transplantation survival, (ii) chemical enhancement of growth and modulation of inhibitory pathological processes and (iii) provision of the physicochemical environment needed for regenerating tissue (e.g., alignment and extracellular matrix cues) [2,3]. In particular, the restoration of aligned neural tissue structures will be important when developing multicellular, tissue-engineered constructs for spinal cord repair.

In this regard, electrospun nanofibres can be readily aligned and they exhibit high porosity and high surface area to promote cell attachment [4]. Moreover, electrospun fibres can be engineered at a nanometre scale which offers a comparative advantage versus bioprinted scaffolds, which only offer micrometre-scale resolution [5]. They are now widely manufactured at the scale for clinical use and have been engineered into 3D constructs for potential implantation. Using this technique, the implantation of a conduit of aligned electrospun fibres on a complete transection of the spinal cord in rats resulted in robust and long-distance axonal regeneration when compared to random fibre controls [6]. Moreover, implantation of an aligned construct of polylactic acid fibres on injured spinal cord organotypic slices promoted growth of aligned nerve fibres and astrocytes within the lesion gap following fibre direction [7]. Such fibres have also been reported to allow incorporation and slow release of molecules of varying sizes (e.g.: NT-3 and miR-222) for SCI treatment, enhancing axonal regeneration in rats [8].

The potential to engineer pre-aligned, stem cell implants has also been demonstrated. For example, NSCs grown and differentiated on aligned electrospun nanofibers generated neurons which aligned at ≤10° of the fibre direction [9,10]. In addition, neurite length was reported to be higher on aligned fibre constructs than on random fibre constructs [11]. Seeding of astrocytes and oligodendrocyte precursor cells (OPCs) on aligned fibres resulted in alignment and elongation of both cell types along the fibres [12]. Further, the functionalization of aligned fibres with a peptide sequence found in laminin allowed for cell survival and alignment of neurons with the fibres. Mouse embryonic stem cells (mESC) expressed mature neuronal markers at earlier time points on electropsun fibres than when cultured on laminin coated coverslips [13]. Similarly, mESCs grown on aligned fibres differentiated towards a neuronal phenotype at a higher rate than when the mESC were seeded on random fibres or a gelatin control [14]. 

These recent advances in the field highlight the strong clinical potential and feasibility of engineering a highly aligned, multifunctional stem cell implant. However, current research has relied on the use of cell lines or ESC-derived stem/progenitor cells. Such cell lines have inherent risks associated with teratoma formation, especially if residual ESCs are present, and they lack robust and reproducible differentiation protocols [15,16]. Further, there is limited systematic assessment of both the alignment of stem/progenitor cells and the subsequent alignment of their daughter cell progeny on aligned electrospun nanofibers. An initial stem cell analysis will provide alignment data in the scaffold pre-implantation. An assessment of differentiated cell alignment within the implant will provide information on how encapsulated cells will behave post-implantation within the implant and also how surrounding endogenous neural cells may interact with the implant. This is vital as aligning neurite growth in sites of SCI has been shown to facilitate repair [6]. Also, it has been repeatedly shown that aligned astrocytes can encourage and guide neurite growth to potentially improve regeneration [17,18,19]. Finally, to date, there is also no established robust methodology to statistically compare alignment between biological repeats with results often judged qualitatively. Given the drive towards scalable, reproducible manufacture of tissue-engineered constructs, the latter is a vital tool for future commercial development.

Therefore, this study aimed to (i) systematically assess alignment of primary cultures of NSCs and differentiated progeny to electrospun nanofibres; (ii) develop a reproducible quantification strategy to characterise alignment in fibres and associated alignment in neural cells and (iii) examine the broader applicability of the approach by investigating scaffold-mediated alignment in another primary population of progenitor cells, the oligodendrocyte progenitor cells (OPCs).

## 2. Methodology

**Preparation and functionalisation of Cellevate^TM^ fibres.** To examine whether neural cells aligned to aligned nanofibers, we employed pre-fabricated aligned and random nanofiber scaffolds for comparison. Glass coverslips were also used to ensure that nanofibers did not affect cell viability, proliferation and differentiation from standard culture conditions. 3D-NanoMatrix^TM^ 24-well plates with randomly distributed or aligned fibres were kindly donated by Cellevate^TM^ (Lund, Sweden). The fibres provided by the company were delivered in individual inserts placed in 24-well plates, with each insert consisting of a plastic ring tensing all the edges of the fibres. The fibres are made of biodegradable poly-u03b5-caprolactone (PCL) and have a diameter of ca. 700 nm (Cellevate^TM^—personal communication). The necessary inserts were transferred to an empty 24-well plate for each biological repeat. Unless stated otherwise, glass coverslips, random and aligned fibres were washed with 70% ethanol for 5 min. The ethanol was rinsed with water and the materials were incubated at 37 °C with poly-ornithine (1:5) (P4957, Sigma, Dorset, UK) for 1 h. Then, they were washed with water and incubated again with laminin (1:200) (Sigma L2020) for 1 h. Finally, they were washed three times with water and stored in the incubator until use.

**NSC culture setup and maintenance.** Brains from P0–P3 mice were dissected and kept in sterile phosphate buffered saline (PBS) on ice. Under a dissection microscope, the sub-ventricular zone was isolated and kept in Neurosphere Medium (NSM) (3:1 mix of DMEM (41966029, Fisher, Loughborough, UK), F-12 (Fisher 21765-029) supplemented with: B-27 (2%) (Fisher VX17504044), bFGF (20 ng/mL) (100-18B, Peprotech, London, UK) and EGF (20 ng/mL) (RnD Systems 236-EG-200), Heparin (5 µg/mL) (Sigma H3149), Pen-strep (50 U/mL of penicillin, 50 µg/mL of streptomycin) (Fisher 11528876)). The tissue was dissociated with 100 µL of DNAse I (Sigma 10104159001) for every four brains. The cell solution was centrifuged (5 min, 1000 rpm) and passed through a cell strainer (Fisher 22363547). Finally, the density of live cells was counted using trypan blue (Sigma T8154) and cultured in T25 flasks in 5 mL of NSM at a density of 1 × 10^5^ cells/mL and kept in an incubator in 5% CO_2_ at 37 °C. Two days post-dissection, 5 mL of NSM were added to the flasks for feeding purposes. NSCs were allowed to proliferate as neurospheres and the flasks were fed every other day with a 50% medium change.

After 5 to 7 days, the neurospheres were collected in a falcon tube and dissociated by TrypLE incubation for 5 min. Then, they were centrifuged (5 min, 1000 rpm) and the pellet was dislodged and passed through a cell strainer. Finally, trypan blue-stained cells were counted with a hemocytometer and seeded on the fibres and glass coverslips in 600 µL of monolayer medium (MLM) (1:1 mix of DMEM: F-12 supplemented by, N2 (1%) (Fisher VX17502048), bFGF (20 ng/mL), EGF (20 ng/mL), Heparin (5 µg/mL), Pen-strep (50 U/mL of penicillin, 50 µg/mL streptomycin)) at a density of 1.5 × 10^5^ live cells/mL. The cells were fed every other day with a 50% medium change.

NSCs were allowed to proliferate for 5 days before being fixed with 4% paraformaldehyde (PFA) for 30 min or allowed to differentiate to astrocytes, neurons and oligodendrocytes by swapping the medium to differentiation medium (Diff-M) (3:1 mix of DMEM: F-12 supplemented by: B-27 (2%), Heparin (5 µg/mL), Pen-strep (50 U/mL of penicillin, 50 µg/mL streptomycin) and FBS (1%)). The latter were kept in the incubator for a further 7 days, with feeding every 2–3 days, before being fixed with 4% PFA. 

**OPC culture setup and maintenance**. Mixed glial cultures for the generation of OPCs were derived from disaggregated cortices of CD1 mice litters between postnatal day 1–3. The dissection procedure to extract cortices was followed as described by McCarthy and De Vellis (1980) [20] and Chen et al., (2007) [21]. This procedure involved mincing the cortices into 2–3 mm pieces with a sterile scalpel, which were then transferred with D10 medium (DMEM, 1 mM of sodium pyruvate (Sigma S8636), 2 mM of glutaMAX (Fisher 35050), 10% FBS, 1% Pen-Strep) into a falcon tube. The tissue was then triturated 40 times with a sterile Pasteur pipette, followed by further trituration and mechanical dissociation with 21 G and 23 G hypodermic needles (3 times each). The cells were centrifuged at 1000× *g* rpm for 4 min, followed by resuspension in D10, with the suspension then filtered through 70 µm and 40 µm cell strainers. Cell viability was assessed before plating. 

T175 flasks were coated in 10 μg/mL of poly-D-lysine (PDL) for 20 min, followed by 3 consecutive washes in sterile dH20 at 5 min intervals. Next, cells were diluted in D10 at a density of 2 × 10^5^ cells/mL in 20 mL and added to each flask. Mixed glial cultures were maintained with D10 medium and a 50% change of medium was conducted every 2–3 days. Cultures were kept in a humidified incubator at 37 °C in an atmosphere of 5% CO_2_ and 95% humidified air. The mixed glial cultures were initially cultured for 10 days to form a stratified cell layer of basal astrocytes, an intermediate layer of OPCs and lastly, a top layer of microglia.

Sequentially shaking the culture flasks after 10 days allowed for the isolation of purified OPC cultures. Prior to the isolation of OPCs, mechanical shaking of the mixed glial culture flasks for 2 h on an orbital shaker at 220 rpm detached the loosely adherent, surface microglia. Next, the culture flasks were incubated at 37 °C (5% CO_2_/95% humidified air) for a minimum of 2–3 h to restore the physiological pH. Following this, flasks were sealed with a double layer of parafilm to prevent gas escape and were placed on the orbital shaker at 220 rpm for 16–18 h, to allow enough time for the OPCs to detach from the astrocyte bed layer. OPCs were then pooled and centrifuged at 1500 rpm for 6 min and were resuspended in OPC maintenance medium (DMEM, 2 mM of glutaMAX, 1 mM of sodium pyruvate, 50 U/mL of penicillin and 50 U/mL of streptomycin, 03.1% bovine serum, 5 μg/mL of insulin, 50 μg/mL of transferrin, 10 nM of biotin, 30 nM of sodium selenite, 10 ng/mL of basic fibroblast growth factor (FGF2) and 10 ng/mL of platelet-derived growth factor (PDGF-aa) (Peprotech).

A cell count was then conducted and the OPCs were seeded onto aligned and random fibre Cellevate^TM^ constructs at a density of 2.5 × 10^5^ cells/mL in 300 μL of OPC maintenance medium. The plates were left at 37 °C (5% CO_2_ and 95% humidified air) for 1 h to allow for initial cell attachment. An additional 300 μL of OPC medium was then added to make a total of 600 μL per well. Cells were kept in the incubator for 3 days before fixing.

**Proliferation assay.** Proliferative cells were incubated with EdU from the Alexa Fluor 594 kit (Invitrogen 10410845) for 6 h before fixing with 4% PFA and later stained following the Invitrogen Click-iT^®^ EdU Imaging Kits Handbook protocol.

**Survival assay.** Cells were incubated for 20 min with ethidium homodimer-1 (Sigma Aldrich E1903) (6 µM), calcein-AM (VWR 80011-1) (4 µM) and DAPI (2 µg/mL) diluted in DMEM. After incubation, cells were washed once with PBS and imaged live. 

**Immunocytochemistry.** After fixing, cells were washed 3 times for 5 min in PBS and then incubated for 30 min with blocking solution (PBS + 0.3% Triton X-100 (Sigma T9284) + 10% Normal Donkey Serum (Jackson ImmunoResearch 017-000-121)). Following this, cells were incubated at 4 °C overnight with the appropriate primary antibody [Nestin (1:200, 611658, BDbiosciences, Franklin Lakes, NJ, USA) and Sox-2 (1:1000, Sigma Aldrich AB5603) for NSCs, GFAP (1:500, 644702, BioLegend, San Diego, CA, USA) for astrocytes, Tuj-1 (1:500, BioLegend 802001) for neurons, MBP (1:200, 160223, BioRad, Hercules, CA, USA) for oligodendrocytes or NG2 (1:200, AB5320, Millipore, Burlington, MA, USA) for OPCs] suspended in blocking solution. The next day, the cells were incubated with the appropriate secondary antibodies (1:200) + DAPI (2 µg/mL) in blocking solution for 2 h at room temperature in the dark and then washed 3 times, 5 min/wash. Finally, the glass coverslips and fibres were mounted on glass slides with mounting medium (Vectashield H-100, Vector Laboratories, Burlingame, CA, USA) and covered with a glass coverslip.

**Scanning Electron Microscopy (SEM)**. For a high-resolution and detailed imaging of the cell-laden scaffolds, some samples were fixed with 2.5% glutaraldehyde diluted in 0.1 M sodium cacodylate (CAC) in preparation for SEM. Then, these samples were processed with the OTOTO protocol established by Fernandes et al. (2015) [22] and mounted on SEM stubs. To enhance conductivity, the edges of the coverslips were coated with silver paint before imaging. SEM imaging was carried out at the standard setting of 5 Kv and images were taken at ×1.5 k magnification.

**Image acquisition and analysis.** NSC and daughter cell cultures were imaged under a fluorescence microscope (Leica, Wetzlar, Germany, DMC 2500 LED) equipped with a CCD camera (DFC350 FX). The software used for imaging was Leica Application Suite X v.1 (2017). OPC enriched cell culture images were acquired as a z-stack with an Axio Observer.Z1 microscope equipped with an AxioCam MRm powered by Zen 2 (blue edition) software (Carl Zeiss MicroImaging GmbH, Goettingen, Germany). Z-stack CZI images were deconvolved with Huygens Professional version 19.04 (Scientific Volume Imaging, The Netherlands, http://svi.nl), using the CMLE algorithm, with signal-to-noise ratio (SNR):40 and maximum 50 iterations.

In all cases, images from 3 randomly selected fields were taken from each sample. DAPI staining was used to count the total amount of cells for each field and compare it to the appropriate staining (antibody, EdU or ethidium homodimer-1). For each sample, the results from each field were added and the percentage of antibody positive cells was calculated. For each experimental group, at least 100 cells were counted in each biological repeat. The results obtained in each biological repeat were averaged to obtain the final outcome.

To assess cell and fibre orientation for each sample, we employed the “Directionality” plug-in for ImageJ Fiji (https://imagej.net/plugins/directionality, first accessed on 1 September 2018). We then developed additional protocols to further enable us to correlate fibre alignment with cell alignment. Here, for each field, a low magnification fluorescence image and the corresponding bright field image were taken. Fibre alignment was then calculated using the Directionality plugin. The corresponding cellular alignment was then calculated using the counterpart fluorescence image. A Fourier components method was selected as the analytical pathway to calculate the orientation distribution of fibres and cells within the image. Here, a histogram was generated where each structure identified within the image by the software was assigned to bins of each integer angle between −90° and +90°. The alignment of the fibres and cells in each field could then be compared. Three fields were taken per sample. 

To confirm that the images of the aligned constructs showed aligned fibres and that the images of random constructs were completely isotropic, the orientation peak (angle in which more fibres are oriented) of the data acquired from the bright field images was calculated with Excel Office using the formula: IF(AND(A2 > A1, A2 > A3), “PEAK”, “0”). Then, the histogram was shifted so the peak corresponded to 0° (equivalent to rotating the image to obtain horizontal fibres). This process was repeated for each biological repeat and the outcome was averaged and plotted for both random and aligned fibres. To compare the level of alignment between samples, the area under the curve for the orientation peaks (between the angles −30° and 30°) was calculated for both random and aligned fibres in each biological repeat and normalised with respect to the total area under the curve. Then, the results were plotted, tested for normality and analysed with a Wilcoxon test for paired samples.

A similar process was followed to determine cell orientation with respect to the fibres. In this case, the orientation peak was determined on the bright field image for aligned fibres and this peak then shifted to 0°. The orientation data from the corresponding images (fluorescence on aligned fibres, bright field on random fibres and fluorescence on random fibres) was shifted according to the peak on the bright field aligned fibres. The process was repeated for each biological repeat and the resulting data was averaged and plotted as the mean ± standard error of the mean (SEM) in an XY graph.

**Statistical analysis.** All statistical analyses were carried out using GraphPad Prism. All values are expressed as the mean ± SEM unless stated otherwise. All graphs were designed with GraphPad Prism, except the XY directionality graphs, which were designed using ggplot2 in Rstudio. Bar graphs also show individual data points as dots, each dot representing a different biological repeat. For analysis of cell type percentages, cell survival and cell proliferation of a non-Gaussian distribution was assumed for all data sets and a non-parametric Kruskal–Wallis test (with Dunn’s correction for a multiple comparison test) was carried out to test if there were any statistical differences between groups. To analyse the correlation coefficient of the cell orientation versus the fibre orientation, the data sets were plotted in a linear graph with the fibre orientation values in the X axis and the cell orientation values in the Y axis. A two-tailed Spearman correlation test with a 95% confidence interval was carried out. Each mouse litter was counted as one biological repeat.

**Ethical statement.** All animal use was in accordance with the Animals (Scientific Procedures) Act of 1985 (UK). This material has not been published in whole or in part elsewhere. All authors have been personally and actively involved in substantive work leading to the manuscript and will hold themselves jointly and individually responsible for its content.

## 3. Results

### 3.1. Alignment Quantification Methodology Could Distinguish between Aligned and Random Nanofiber Constructs

**Figure 1:**
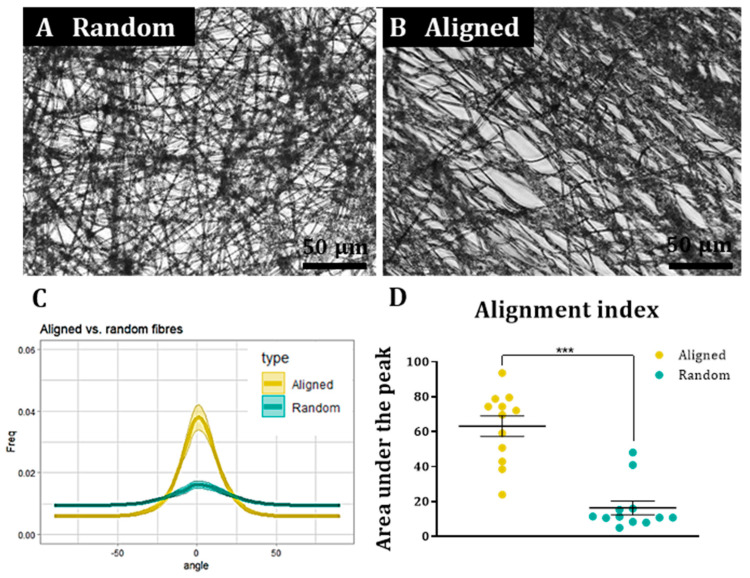
Representative bright field images of random (**A**) and aligned (**B**) fibres with orientation graph showing the fibre directionality peaks (**C**) and comparison of alignment indexes between random and aligned fibres (**D**) (Wilcoxon test for paired samples, n = 12, *** *p* < 0.001).

Phase contrast microscopy revealed matrices of random or aligned nanofibres (Figure 1A,B), forming a compact but porous structure that allows for 3D culture of cells. The computational analysis showed that a high frequency of fibres was oriented in the same direction on the aligned fibres, with a significantly reduced frequency in the random fibres. The percentage of fibres aligned within ±30° of the peak direction was approximately 4-fold higher on aligned fibres (63.2 ± 5.8%) than on random fibres (16.4 ± 3.9%; Figure 1C,D; *p* < 0.001, n = 12).

### 3.2. Quantification Method Demonstrates NSCs Align to Aligned Electrospun Nanofiber Scaffolds

**Figure 2:**
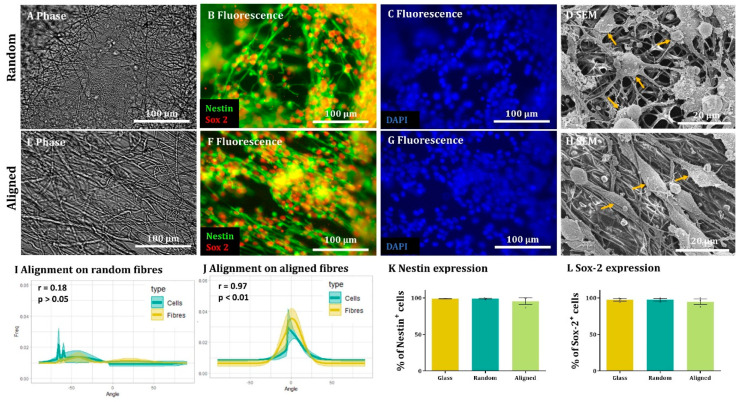
**NSCs align to aligned nanofibers but not random fibres.** (**A**–**C**) Representative images of (**A**) random nanofibers in phase with counterpart fluorescence images showing (**B**) NSCs stained with NSC cytoskeleton marker Nestin (green) and NSC nuclear marker Sox-2 (red) with insets showing alongside (**C**) nuclear marker DAPI (blue). (**D**) Representative SEM images of NSCs grown on random fibres. (**E**–**G**) Representative images of (**E**) aligned nanofibers in phase with counterpart fluorescence images showing (**F**) NSCs stained with Nestin and Sox-alongside (**G**) DAPI. (**H**) Representative SEM images of NSCs grown on aligned fibres. In SEM, images examples of putative NSCs are highlighted by the orangearrows. (**I**,**J**) XY graphs indicating the frequency at which cells and fibres are oriented at each angle, with the halo representing the SEM, on random (**I**) and aligned (**J**) fibres. A correlation analysis revealed low correlation between cells and random fibres (r = 0.1842, *p* > 0.05) and a high correlation between aligned fibres and cells (r = 0.9680, *p* < 0.001; n = 3, two-tailed Spearman correlation test with a 95% confidence interval). (**K**,**L**) Bar graphs represent the percentages of Nestin- (**K**) and Sox-2- (**L**) positive cells on glass, random and aligned substrates with no differences detected (Kruskal–Wallis test, n = 4, *p* > 0.05).

Microscopic visualisation of NSCs seeded on random and aligned fibres showed classical bipolar and occasional multipolar cells, positive for nestin and Sox-2. Nuclei generally displayed rounded morphology with cell distribution and number per field similar across all experimental conditions (Figure 2). On the random fibres, there were no obvious signs of cell orientation. This was markedly different on the aligned fibres, where cell processes were seen to follow the same general direction, apparently along the fibres (Figure 2). Using SEM, we were able to confirm that NSCs extended processes along the fibres and appeared to display contact-mediated orientation with the fibres. This was less apparent in the random fibres, where cell processes did not appear oriented in a particular direction and also often seemed to grow over multiple fibres, rather than follow a specific fibre (Figure 2D,H). Our computational analysis supported the microscopic observations and showed a pronounced cellular directionality peak on the aligned fibres which was absent in the random fibres. Moreover, we were able to demonstrate a strong correlation between fibre and cell alignment under aligned nanofiber conditions which was absent in random nanofiber conditions (Figure 2I,J). The fibres (random or aligned) did not affect proportions of cells expressing the stem cell markers, with high levels of cells (>95%) positive for nestin and Sox-2 and no significant differences between substrates (Figure 2K,L). 

### 3.3. Daughter Neurons and Astrocytes, but Not Oligodendrocytes, Also Align with the Aligned Nanofibers

**Figure 3:**
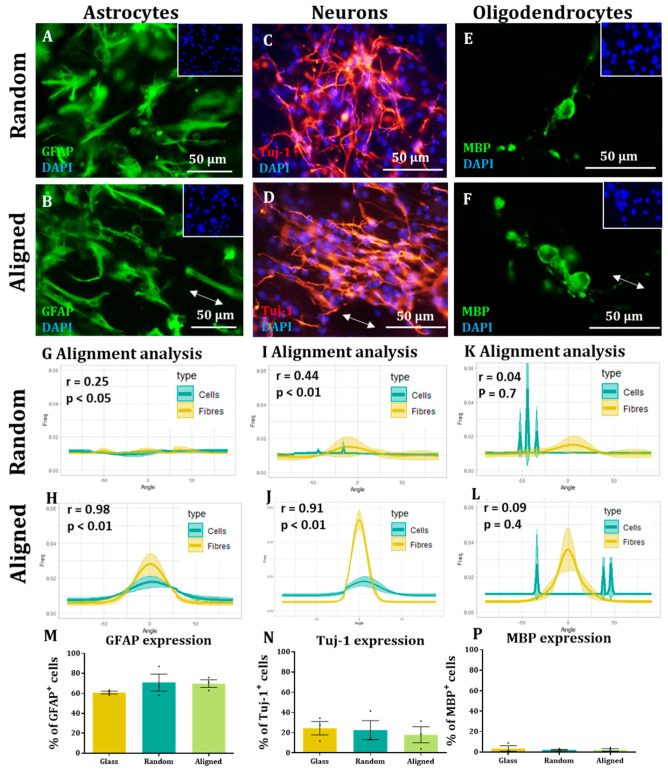
**Neurons and astrocytes retain alignment on aligned scaffolds.** (**A**–**F**) Representative image of astrocytes, neurons and oligodendrocytes on either random or aligned fibres as indicated. Direction of aligned fibres is shown by white arrow. (**G**–**L**) XY graphs indicating the frequency at which cells and fibres are oriented at each angle for each different cell type and condition. Correlation analyses (n = 3, two-tailed Spearman correlation test with a 95% confidence interval) were performed for each cell type, with r and *p* numbers showing strength of correlation. (**M**–**P**) Bar graphs representing the percentages of (**M**) GFAP, (**N**) Tuj-1 and (**P**) MBP positive cells on glass, random and aligned substrates with no differences detected (Kruskal-Wallis test, n = 4, *p* > 0.05).

After differentiation of NSCs on the substrates for seven days, all three major cell types were produced (astrocytes, neurons and oligodendrocytes) across all conditions. Astrocyte morphology appeared more elongated on random fibres than on coverslips (Figure 3A) but cell orientation seemed random. By comparison, numerous instances of astrocyte projections aligning to the direction of the fibres were observed when cultured on the aligned fibre substrates. It should be noted, this was not uniform among all cellular projections, with some cell spreading observed over the fibres and some projections not in alignment with the fibres (Figure 3B). Neurons on random and aligned fibres displayed the classical morphology of immature neurons—a small cell body with singular or multiple neurite projections. On random fibres, these projections appeared to be randomly oriented. However, on the aligned fibres the majority of the projections appeared to follow the direction of the aligned fibres. Similar to the astrocyte projections, there were examples of neurites projecting across the fibre direction also (Figure 3C,D). Oligodendrocytes also displayed classical morphology with a clear, small cell soma and thickened membrane projections, staining positive for MBP. In contrast to the previous cell types, no obvious directionality was observed in these projections on random or aligned fibres (Figure 3E,F). Lower-magnification images depicting fibre orientation and fluorescence images of encapsulated cells are available in Supplementary Figure S1. Microscopic observations were strongly supported by the computational analysis. No obvious directionality was detected for any cell type on the random fibres, with no correlation between fibre direction and cell direction. Clear peaks of direction were observed for the astrocytes and neurons grown on aligned fibres. Further, their direction was strongly correlated with that of the fibres (Figure 3G–L). No substrates were found to significantly impact differentiation with proportions of cells expressing GFAP, Tuj-1 or MBP similar across all conditions (Figure 3M–P).

### 3.4. Nanofibre Scaffolds Did Not Demonstrate Adverse Effects on the Health of NSCs or Their Daughter Cell Populations

**Figure 4:**
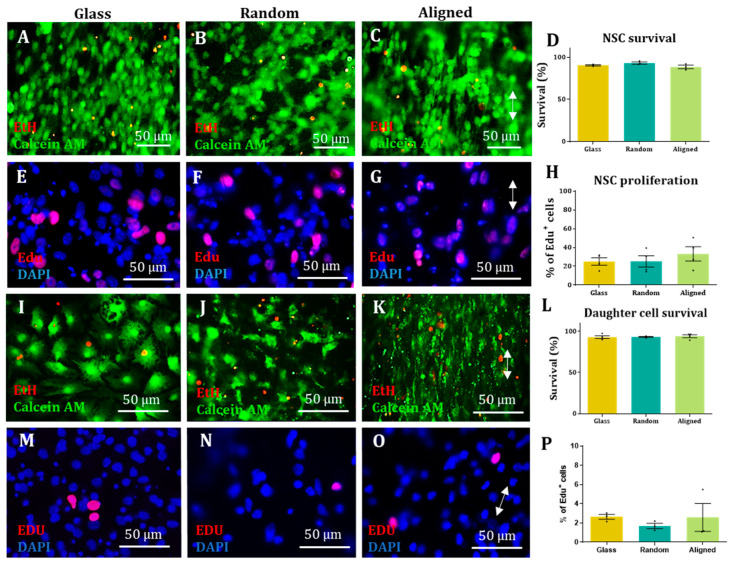
**NSC and daughter cell health is retained on electrospun nanofiber scaffolds.** (**A**–**D**): Representative images of NSCs stained with calcein AM, indicating live cells (green) and ethidium homodimer-1 (EtH), indicating dead cell nuclei (red) on glass (**A**) random fibres (**B**) and aligned fibres (**C**; arrow indicating fibre direction) and graphical representation of survival rates (Kruskal–Wallis test, n = 3, *p* > 0.05) (**D**). (**E**–**H**) Representative images of NSCs stained with DAPI, a cell nuclear marker (blue) and EdU, a proliferative cell nuclei marker (red) on glass (**E**), random fibres (**F**) and aligned fibres (**G**) with graphical representation of proliferation rates (Kruskal–Wallis test, n = 4, *p* > 0.05) (**H**). (**I**–**L**) Representative images of live/dead staining of daughter cells on glass (**I**) random fibres (**J**) and aligned fibres (**K**; arrow indicating fibre direction) and graphical representation of survival rates (Kruskal–Wallis test, n = 3, *p* > 0.05) (**L**). (**M**–**P**) Representative images of daughter cells stained with EdU on glass (**M**), random fibres (**N**) and aligned fibres (**O**; arrow indicating fibre direction) with graphical representation of proliferation rates (Kruskal–Wallis test, n = 4, *p* > 0.05) (**P**).

All cell types grown on the scaffolds demonstrated classical morphologies, with no apparent detriment to cell structure (i.e., cell staining appeared in line with expected staining patterns and cell profiles). In terms of cell health, live/dead staining revealed over 90% NSC survival across all conditions after five days in vitro (Figure 4A–C), with no statistical differences between groups (Figure 4D). In addition, a proliferation assay also revealed no statistical differences in proliferation rate across conditions (Figure 4E–H). After differentiation, over 90% of the cell population was viable, with no statistical differences between groups (Figure 4I–L). A proliferation assay on differentiated cell populations revealed that approximately 2% of the cell population stained positive for the proliferation marker, EdU, with no statistical differences between groups (Figure 4M–P).

### 3.5. The Quantification Method Also Demonstrates That the Key Transplant Population of OPCs Are Guided by Aligned Electrospun Nanofiber Scaffolds

**Figure 5:**
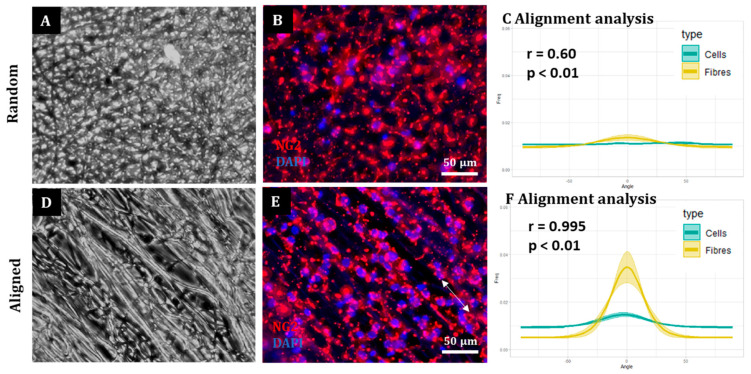
**OPCs align to electrospun aligned fibres.** Representative fluorescence images of OPCs on (**A**,**B**) random and (**D**,**E**) aligned fibres. Arrow in (**E**) indicates fibre direction. (**C**,**F**) Graphs representing the mean (±SEM) fibre and cell orientation of OPCs on random (**C**) and aligned (**F**) fibres. Correlation results are also displayed with the associated r and p numbers on the graph (n = 3, two-tailed Spearman correlation test with a 95% confidence interval).

Finally, a population of OPCs was also successfully cultured on random and aligned fibres (Figure 5). The majority (>80%) of cells were positive for the OPC marker NG-2. Microscopic analysis indicated that OPCs on random fibres appeared randomly oriented (Figure 5A,B), which was supported by a flat directionality curve for both fibres and cells and a weak correlation coefficient (Figure 5C). In contrast, OPCs appeared to grow along aligned fibres (Figure 5D,E) with nuclei appearing to line up on the fibres and projections extending in the same direction of the fibre direction. Computational analysis indicated a relatively small peak in cell directionality, although this was strongly positively correlated with the fibre direction (Figure 5F). 

## 4. Discussion

Here, we show that alignment of multiple neural cells incorporated in tissue-engineered constructs can be reproducibly and quantitatively analysed through the use of a freely available directionality plugin, combined with data rotation and a correlation test. Biological repeats could be combined into the analysis, allowing statistical analysis of alignment and comparison between different scaffold conditions (e.g., aligned versus random scaffolds). Therefore, this could be used as a reproducible and comparative analytical method by neural tissue-engineering laboratories worldwide. Moreover, using our technique, we were able to robustly demonstrate that NSCs and OPCs aligned to aligned electrospun nanofibers. Further, the quantification data showed that astrocytes and neurons born from NSCs retained some alignment to the aligned fibres. Our data show that a pre-aligned stem cell-laden construct could be developed for clinical applications, and this alignment conformation is retained as the cells in the implant mature.

The computational analysis of nanofiber and cell alignment appear particularly useful for future development of aligned neuro-cellular regenerative implants. Whilst previous studies have provided evidence of cellular alignment to aligned scaffolds, these are often qualitative [11,23] or use just one sample for a quantitative analysis, without comparison between biological repeats [13]. Alternatively, other studies have quantified the cell’s angle deviation from the fibre they are aligned to [9,24], resulting in repetitive and time-consuming work, with risk of error. Our high-throughput analytical method allows for an unbiased, reliable analysis of cell alignment of multiple cells simultaneously with comparison between biological repeats. This means that variables which could affect alignment (e.g., nanofiber density, diameter, chemistry) can be rapidly and consistently assessed. The methodology is versatile, as demonstrated by application to a range of neural cells, but we also believe the method can be applied to other aligned scaffolds. In a separate unpublished study, we have now applied the same technique to show that aligned pores extending through 3D cellulose scaffolds also impart alignment over NSCs grown in the 3D scaffolds. 

To the best of our knowledge, this is also the first report investigating both parent stem cell alignment, and subsequent daughter cell alignment following the differentiation of aligned, attached stem cells. We show the potential of an aligned implant of NSCs to differentiate into repair mediating cell populations, also showing alignment. This may be vital for clinical application as astrocyte alignment has been shown to induce higher neurite outgrowth and neuronal guidance [19], with previous studies also suggesting thatneurites follow aligned astrocytes [7]. Alignment of neurites in areas of SCI is well documented to improve regeneration [6], but aligning neurons born from transplanted stem cells may also be important in repairing short-distance, neural circuits in neurological injury [25]. Whether the cells retain alignment throughout the complex differentiation process or lose connection with the fibres then re-establish alignment is not currently known. Presumably the original stem cell populations adhere to the fibre coating through cell adhesion molecules, with pre-coating fibres previously proven to be vital for cell extension along fibres [12]. However, it can be predicted that as the cell changes its proteomic profile, these adhesion molecules would be turned over. Further investigation into this phenomenon could inform future design of nanofiber chemistry to allow cell attachment throughout differentiation. 

We were not able to see NSC-derived oligodendrocytes aligned on the fibres. This could be due to the low numbers of these cells present in our cultures or due to the tendency to find these cells sitting on the surface of astrocytes. OPC alignment with electrospun fibres has been observed in co-culture with astrocytes, but similar to our study, the alignment appears to be lost when OPCs differentiated into mature oligodendrocytes [12]. Conversely, we were able to align an enriched population of OPCs on aligned and random fibres. This last experiment indicates the applicability of the methodology described to different neural transplant cell populations, and demonstrates an alternative, aligned glial cell-based implant. In contrast to the other cells described, it is not currently clear whether aligned mature oligodendrocytes would offer improved regeneration over non-aligned oligodendrocytes. Oligodendrocytes extend their membrane to nearby axons and then ensheathe these in myelin to provide conduction. It is tempting to predict then that the other aligned cell types in a multicellular construct can guide regenerating neurites and the neighbouring oligodendrocytes can then provide the necessary myelin, without needing to be an aligned population themselves. 

## 5. Conclusions

We have shown that aligned nanofibers can reproducibly and robustly impart alignment over neural transplant populations, potentially allowing implantation of a pre-aligned cellular scaffold. Further, this alignment can be passed onto the daughter cells, possibly allowing for alignment of regenerating tissue post-implantation. We have also shown how alignment can be assessed across multiple biological repeats, allowing researchers to statistically correlate cell alignment to nanofibers. This may be useful when fine tuning nanofiber production to optimise the parameters to achieve the highest level of alignment. 

## Data Availability

Data is contained within the article or supplementary material.

## Supplementary Materials

The following supporting information can be downloaded at: https://www.mdpi.com/article/10.3390/ma16010124/s1, Figure S1: Representative images showing counterpart phase and fluorescence images of aligned and random nanofibers. Each stain depicts a different cell type in a differentiated population of NSCs as indicated on the figure.
