# Supplementary material for: Systematic Alignment Analysis of Neural Transplant Cells in Electrospun Nanofibre Scaffolds
Source: Materials (Basel). 2022 Dec 23;16(1):124. doi: 10.3390/ma16010124 (PMC9821626; doi:10.3390/ma16010124)
Supplement: Supplementary file 1 [file materials-16-00124-s001.zip › materials-2042848-supplementary.pptx]

## Slide 1
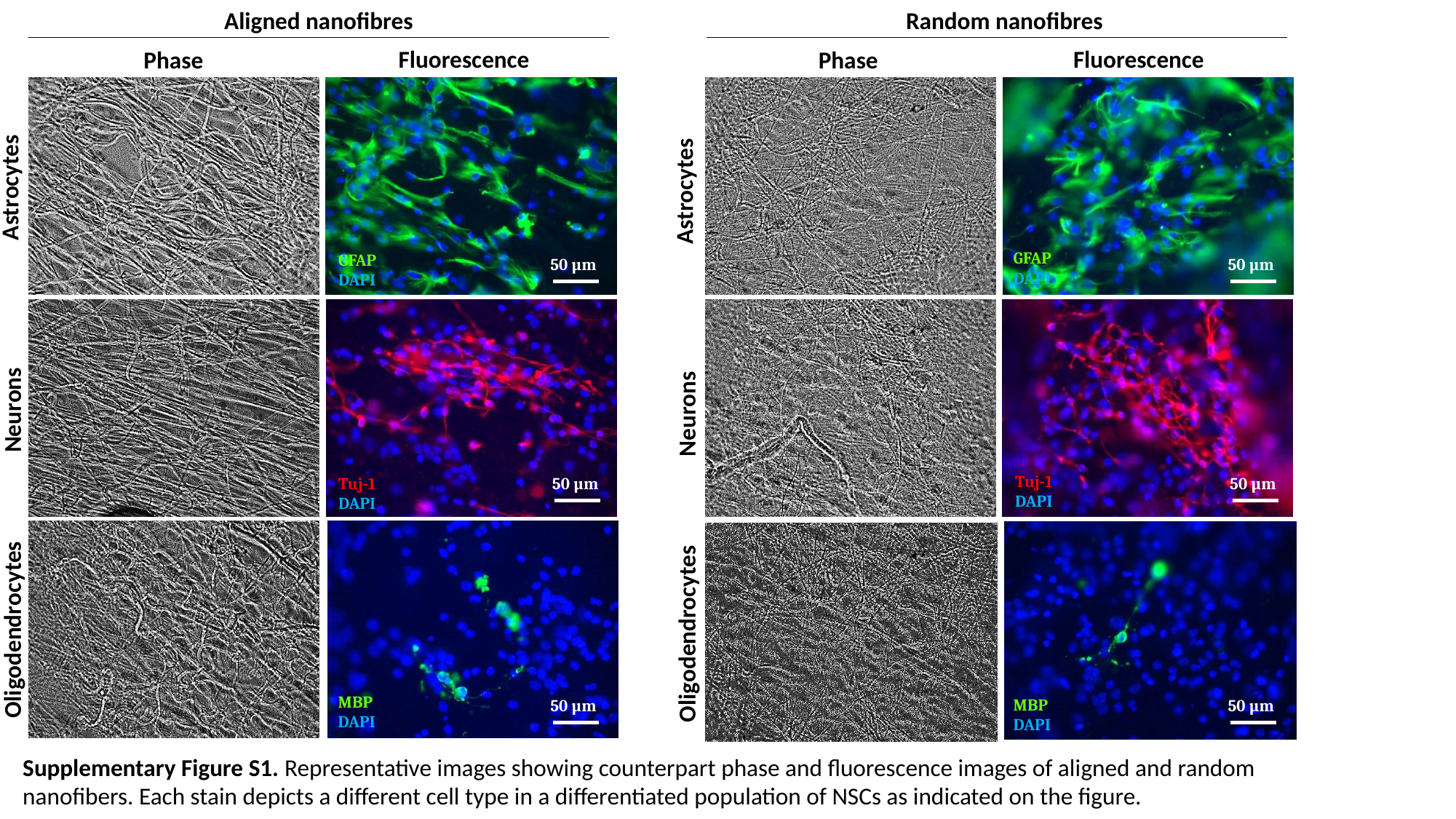

Aligned nanofibres
Random nanofibres
Fluorescence
Fluorescence
Phase
Phase
Astrocytes
Astrocytes
GFAP
DAPI
GFAP
DAPI
50 µm
50 µm
Neurons
Neurons
Tuj-1
DAPI
Tuj-1
DAPI
50 µm
50 µm
Oligodendrocytes
Oligodendrocytes
MBP
DAPI
MBP
DAPI
50 µm
50 µm
Supplementary Figure S1. Representative images showing counterpart phase and fluorescence images of aligned and random nanofibers. Each stain depicts a different cell type in a differentiated population of NSCs as indicated on the figure.
